# A comparative analysis: international variation in PET-CT service provision in oncology—an International Cancer Benchmarking Partnership study

**DOI:** 10.1093/intqhc/mzaa166

**Published:** 2020-12-30

**Authors:** Charlotte Lynch, Irene Reguilon, Deanna L Langer, Damon Lane, Prithwish De, Wai-Lup Wong, Fergus Mckiddie, Andrew Ross, Lorraine Shack, Thida Win, Christopher Marshall, Mona-Eliszabeth Revheim, Bolette Danckert, John Butler, Sabina Dizdarevic, Cheryl Louzado, Canice Mcgivern, Anne Hazlett, Cindy Chew, Martin O’connell, Samantha Harrison

**Affiliations:** International Cancer Benchmarking Partnership (ICBP), Policy & Information, Cancer Research UK, 2 2 Redman Place, London, E20 1JQ, UK; International Cancer Benchmarking Partnership (ICBP), Policy & Information, Cancer Research UK, 2 2 Redman Place, London, E20 1JQ, UK; Brand & Strategy, eConsult Health Ltd, 46-48 East Street, Surrey, KT17 1HQ, UK; Cancer Imaging, Ontario Health (Cancer Care Ontario), 620 University Avenue, Toronto, ON M5G 2L7, Canada; Radiology, Pacific Radiology, 123 Victoria Street, Christchurch Central, Christchurch 8013, New Zealand; Surveillance and Cancer Registry, Ontario Health (Cancer Care Ontario), 620 University Avenue, Toronto, ON M5G 2L7, Canada; Nuclear Medicine, Mount Vernon Hospital, East and North Hertfordshire NHS Trust, Rickmansworth Road, Northwood, HA6 2RN, UK; Nuclear Medicine and PET Department, NHS Grampian, 2 Eday Road, Aberdeen AB15 6RE, UK; Dalhousie Medical School, Dalhousie University, 6299 South Street, Halifax, Nova Scotia, NS B3H 4R2, Canada; Surveillance and Reporting, Alberta Health Services (Cancer Control Alberta), 10030-107 Street NW, Edmonton, Alberta, T5J 3E4, Canada; General and Respiratory Medicine, Lister Hospital, East and North Hertfordshire NHS Trust, Coreys Mill Lane, Stevenage, SG1 4AB, UK; Wales Research and Diagnostic PET Imaging Centre, Cardiff University, Cardiff University School of Medicine Health Park, Cardiff, CF14, 4XN, UK; Division of Radiology and Nuclear Medicine, Oslo University Hospital and Institute of Clinical Medicine, University of Oslo, Pb 4950 Nydalen, Oslo, 0424, Norway; Research Centre, Danish Cancer Society, Strandboulevarden 49, 2100 Kobenhavn, Denmark; International Cancer Benchmarking Partnership (ICBP), Policy & Information, Cancer Research UK, 2 2 Redman Place, London, E20 1JQ, UK; Gynaecology Department, Royal Marsden NHS Foundation Trust, 203 Fulham Road, London, SW3 6JJ, UK; Imaging and Nuclear Medicine, Brighton and Sussex University Hospital Trust, Kemptown, Brighton, BN2 1ES, United Kingdom and Brighton and Sussex Medical School, University of Sussex and Brighton, London Road, Brighton, BN1 4GE, UK; Strategy Implementation Planning & Partner Relations, Canadian Partnership Against Cancer, 145 King St, Toronto, ON M5H 1J8, Canada; Department of Regional Medical Physics, Belfast Health and Social Care Trust, 83 Shankill Road, Belfast, BT13 1FD, UK; Department of Regional Medical Physics, Belfast Health and Social Care Trust, 83 Shankill Road, Belfast, BT13 1FD, UK; School of Medicine, Dentistry and Nursing, University of Glasgow, University Avenue, Glasgow, G12 8QQ, UK; Radiology, Mater Misericordiae University Hospital, Eccles Street, Dublin, DO7 R2WY, Ireland; International Cancer Benchmarking Partnership (ICBP), Policy & Information, Cancer Research UK, 2 2 Redman Place, London, E20 1JQ, UK

**Keywords:** cancers, benchmarking, healthcare system, appropriate healthcare, diagnostics

## Abstract

**Objective:**

To explore differences in position emission tomography-computed tomography (PET-CT) service provision internationally to further understand the impact variation may have upon cancer services. To identify areas of further exploration for researchers and policymakers to optimize PET-CT services and improve the quality of cancer services.

**Design:**

Comparative analysis using data based on pre-defined PET-CT service metrics from PET-CT stakeholders across seven countries. This was further informed via document analysis of clinical indication guidance and expert consensus through round-table discussions of relevant PET-CT stakeholders. Descriptive comparative analyses were produced on use, capacity and indication guidance for PET-CT services between jurisdictions.

**Setting:**

PET-CT services across 21 jurisdictions in seven countries (Australia, Denmark, Canada, Ireland, New Zealand, Norway and the UK).

**Participants:**

None.

**Intervention(s):**

None.

**Main Outcome Measure(s):**

None.

**Results:**

PET-CT service provision has grown over the period 2006–2017, but scale of increase in capacity and demand is variable. Clinical indication guidance varied across countries, particularly for small-cell lung cancer staging and the specific acknowledgement of gastric cancer within oesophagogastric cancers. There is limited and inconsistent data capture, coding, accessibility and availability of PET-CT activity across countries studied.

**Conclusions:**

Variation in PET-CT scanner quantity, acquisition over time and guidance upon use exists internationally. There is a lack of routinely captured and accessible PET-CT data across the International Cancer Benchmarking Partnership countries due to inconsistent data definitions, data linkage issues, uncertain coverage of data and lack of specific coding. This is a barrier in improving the quality of PET-CT services globally. There needs to be greater, richer data capture of diagnostic and staging tools to facilitate learning of best practice and optimize cancer services.

## Introduction

International variation in cancer outcomes persists, which was recently demonstrated by the International Cancer Benchmarking Partnership (ICBP) [1]. One of the many factors influencing this variation may be differences in accessing diagnostic tests. By exploring the service provision of these tests across ICBP countries, we aim to better understand access to quality cancer staging tools and therefore what information is available for cancer treatment planning. This study focuses on ^18^F-FDG (^18^F-fluorodeoxyglucose) position emission tomography-computed tomography (PET-CT) as a largely oncology-specific imaging modality, which has been demonstrated to have a crucial role in identifying the clinical stage in many types of cancer [2]. Across several cancer sites, the sensitivity and specificity of PET-CT has been shown to be 93% and 96%, respectively, compared to 52% and 89% of conventional imaging [3]. There are a variety of radiotracers appropriate for use with PET-CT scanning; however, ^18^F-FDG is this paper’s focus as it is the most commonly used in cancer [4]. To our knowledge, no study to date has explored international differences in PET-CT service provision. In this paper, we describe these differences and consider their potential contribution to observed differences in cancer outcomes.

The introduction of PET-CT into oncological imaging in the early 2000s resulted in a substantial increase in scanner quantity worldwide due to the significant time efficiency and cost-efficient advantages it proposed compared to having two separate modalities [5]. The main clinical benefit of PET-CT lies in its ability to link changes in metabolic activity with anatomical imaging, allowing for accurate identification of the location, size and shape of tumours through identifying abnormal cellular activity [4]. The superiority of PET-CT over using CT alone in certain cancers for diagnosis, staging, evaluating metastatic spread, optimizing and monitoring treatment, and assessing prognosis has been demonstrated in the literature [2, 3].

Evidence exists that PET-CT can accurately change the staging of cancers and influence treatment and management strategies [4, 6]. Through accurate staging, PET-CT can reduce unnecessary therapies and surgeries and enable more tailored treatment planning to best suit individual patients (see Appendix and Table 1 for focus on non-small-cell lung cancer (NSCLC) evidence).

Appropriate use of PET-CT services, directed through available clinically approved indications, has implications for clinical effectiveness, cost-effectiveness and patient experience of care. Several economic reviews have shown PET-CT to be cost-effective when used in staging for NSCLC, diagnosing single pulmonary nodules (SPN), restaging after recurrence of colorectal cancer (CRC), staging of lymphoma and detection of distant metastases in head and neck cancer [5].

The diagnostic interval is a complex part of the cancer patient pathway, and various elements of it can affect how swiftly patients move through healthcare systems. Some studies have shown increased mortality and late stage at diagnosis to possibly be associated with longer diagnostic intervals [7, 8]. The impact of different levels of access to PET-CT on the length of diagnostic intervals and cancer outcomes is currently unknown. However, access to diagnostic tests for healthcare professionals and patients can contribute to how timely referrals, investigations and treatments are, as well as the accuracy of the diagnosis and clinical staging [9]. Disparities in PET-CT service provision may therefore contribute to differences seen in cancer outcomes internationally.

Despite the evidence base supporting the use of PET-CT in cancer management, the capacity and quality of PET-CT provision can be restricted by factors including funding, approved clinical indications, workforce, geography and access to radiotracers [10]. International comparisons of these services can allow health systems to learn from one another’s best practice to enhance PET-CT service delivery and possibly patient outcomes.

Improving quality of care, preventing diagnostic and treatment errors and decreasing healthcare costs all rely on learnings from high-quality data [11]. Due to varying data guidelines and practices between countries, there is a lack of uniform diagnostic data collection and coding practices internationally and a lack of previous literature exploring international PET-CT service provision. In this study, we aim to provide an understanding of PET-CT service provision in ICBP countries and recommend ways to optimize PET-CT service provision and cancer care globally.

## Methods

This study focuses on ICBP countries (Australia, Canada, Denmark, Ireland, New Zealand, Norway and the UK) and cancer sites (lung, colon, rectal, oesophageal, gastric, ovarian, pancreatic and liver) as determined by the ICBP’s original protocol [1].

We requested data from 2000 to 2017 on service metrics (Table 1) from PET-CT centres across all 21 ICBP jurisdictions (data sources in Appendix and Table 2).

**Table 1:** ICBP PET-CT access metrics and definitions

Access metric	Definition
Location	Full postal address of every PET-CT scanner used for clinical work, accounting for additional scanners at same location
Date of acquisition	Date, month and year the scanner was bought/acquired
Date of operational start	Date, month and year the PET-CT scanner became operational
Private/public location	Indication of PET-CT being at a public or private location of facility
Median wait time from referral to PET-CT scan	Time taken from patient being referred by clinician for PET-CT to the date scan was performed
Median wait time from PET-CT scan to reporting of results	Time taken from PET-CT scan being performed to the scan results being available and sent to the clinician who referred the patient
PET-CT scans performed annually for all purposes	Number of PET-CT scans performed regardless of purpose, i.e. including those not oncology-specific
PET-CT scans performed annually for oncology purposes	Number of scans performed in cancer patients by cancer site per year
PET-CT scans per indication	Percentage of PET-CT scans carried out per year per indication (diagnosis, staging and surveillance)
Price of scanner	Average price of PET-CT scanner
Price per PET-CT scan	Include (where possible) considerations when calculating cost of each scan, e.g. workforce, cost of machinery and overheads
Price per ^18^F-FDG dose	Include when this price may have changed, e.g. due to changes in demand or urgency

PET-CT stakeholders sourced publicly available administrative data from within their respective jurisdictions. Significant variation was seen in the availability and accessibility of requested data metrics, and so, the analysis focused on the most complete data (see ‘Results’ section).

PET-CT clinical indication evidence was sourced through 51 indication guidance documents, validated by our stakeholders, or regulatory bodies including but not limited to Royal College of Radiologists, Welsh Health Specialised Services Committee and Medical Benefits Schedule Australia. We focus on lung cancer, CRC and oesophagogastric (OG) cancer, due to the greater availability of data on PET-CT activity within these cancer sites. We present PET-CT indications dependent upon their primary purpose: diagnosis of SPN—lung cancer only, staging, treatment planning, treatment assessment and evaluating recurrence.

A working group was formed, with stakeholders from each jurisdiction. Member expertise included clinical radiology, service management and data specialists. Four round-table discussions were held to provide local intelligence around the barriers to PET-CT service delivery and indication development.

Descriptive comparative analyses of use, capacity and indication guidance for PET-CT services between jurisdictions were performed.

## Results

Data capture and availability was variable and limited across ICBP jurisdictions (Appendix and Table 3). Results and jurisdictional comparisons were made and presented based upon available/accessible data. Significant growth in PET-CT scanners was experienced in all ICBP jurisdictions since the early 2000s (Table 2). Variation exists in the capacity, use and indication guidance of PET-CT services across jurisdictions.

**Table 2:** PET-CT scanner quantity in ICBP jurisdictions (2017)

Jurisdiction	N PET-CT scanners^a^^,^^b^	N scanners per 100 000^a^^,^^b^
Australia^c^	46	0.27
Western Australia	7	0.27
Victoria	17	0.27
New South Wales	22	0.28
Canada	51	0.13
New Brunswick	2	0.26
Prince Edward Island	0	N/A
Ontario	13	0.09
Nova Scotia	1	0.10
Saskatchewan	1	0.09
Alberta	4	0.09
Denmark	38	0.66
Ireland	7	0.15
New Zealand	5	0.10
Norway	10	0.19
UK	54	0.08
England	46	0.08
Northern Ireland	1	0.05
Wales	1.2	0.04
Scotland	5	0.09

N/A = Not applicable.

^a^ Figures correct as of 2017—capacity may have increased since then.

^b^ May include scanners dedicated for other uses outside of oncology (e.g. dedicated cardiovascular scanners) and also scanners functioning part-time.

^c^ For three Australian jurisdictions combined; exact number of scanners for whole country not available.

### Variation in data capture, accessibility and availability

Data were available from 17 jurisdictions on number and location of scanner centres; four jurisdictions were excluded from the study. Eleven jurisdictions were able to provide some data on scans per cancer site, but this varied greatly between jurisdictions in the years covered. Reported PET-CT stakeholder access to data on PET-CT time intervals from referral to results was strictly limited and therefore not analysed. Data for cost of scanners, scan and ^18^F-FDG dose were similarly limited and excluded from analysis.

### Variation in scanner quantity and acquisition over time

The number of PET-CT scanners (2017) ranged from 0.04 per 100 000 in Wales to 0.66 per 100 000 in Denmark (Table 2). Prince Edward Island (PEI) in Canada did not have any PET-CT scanners and instead refers patients out of province—likely due to small population size (150 566 in 2017).

Full-time equivalent (FTE—ratio of scanner activity as total hours per week) data were incorporated where known, with total number of scanners being represented as a fraction where appropriate.

**Figure 1:**
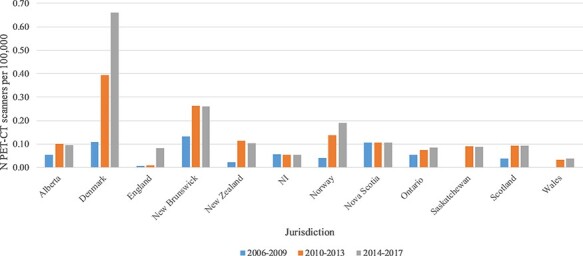
Acquisition of PET-CT scanners (per 100 000 persons) 2006–2017. N.B no dates of acquisition for Australia or Ireland; only Alliance Medical data provided for England.

Acquisition of scanners over time showed the greatest increase in Denmark: 0.02 scanners per 100 000 in 2007, to 0.66 per 100 000 in 2017 (Figure 1). New Brunswick and Norway followed, with an increase of 0.13 and 0.15 scanners per 100 000 over the period 2006–2017. Ten out of 12 jurisdictions with available dates of acquisition installed their first scanners in 2006–2009 period, with Wales and Saskatchewan not acquiring their first scanner until the 2010–2013 period.

### Variation in indication guidance

Variation in how indications for PET-CT are developed and recorded was identified (Table 3). It should be noted that lack of indication guidance does not necessarily reflect what is happening in practice i.e. in Denmark, there appear to be fewer confirmed indications, however, there is flexibility to refer patients outside of these indications (M. Achiam, personal communication, November 2019).

**Table 3:** PET-CT indications for lung, CRC and oesophagogastric cancers across ICBP jurisdictions

		Lung						CRC						Oesophagogastric^a^					
Jurisdiction	S	RS	AS	Su	TP	TA	RE	S	RS	Su	TP	TA	RE	S	RS	M	TP	TA	RE
Alberta	•	•	•	•	•	•	•		•	•	•		•	•	•	•	•		•
Australia	•		•		•	•	•		•		•		•	•			•	•	•
Denmark	•				•				•		•	•	•	•					
England	•		•		•	•	•		•		•	•	•	•			•	•	•
Ireland	•		•		•		•		•		•	•	•	•			•	•	•
New Zealand	•		•		•				•		•	•	•	•			•		
Norway	•		•		•		•							•	•		•	•	•
Nova Scotia	•		•	•									•	•	•	•			
Northern Ireland	•		•			•			•		•	•	•	•					
Ontario	•	•	•		•		•		•		•		•	•	•			•	•
Saskatchewan	•		•		•				•						•		•	•	
Scotland	•		•		•		•				•		•	•			•		•
Wales	•		•		•		•		•					•			•		

S = Staging; RS = Restaging; AS = Assessment of solitary pulmonary nodule; Su = Surveillance; TP = Treatment planning^b^; TA = Treatment assessment; RE = Recurrence evaluation.

^a^ primarily focused on oesophageal and GOJ tumours; evidence is more varied and emerging for gastric cancers.

^b^ Treatment planning refers to aiding guidance upon treatment decision e.g. chemotherapy or surgery.

All jurisdictions use PET-CT for primarily oncological purposes. Other non-oncology indications (cardiology, neurology and inflammatory conditions) contribute less to use. PEI and New Brunswick were not included in Table 3. No publicly available PET-CT indications could be sourced for New Brunswick. PEI outsources its referrals to other provinces due to it not having a scanner; referrals are approved based on the centre receiving referral.

Table 3 shows a comparison of the specific uses recommended for PET-CT for lung, CRC and OG cancers, with Table 4 demonstrating key differences/similarities between jurisdictions. Full details of indication guidelines are shown in Appendix and Table 4.

**Table 4:** Notable comparisons between ICBP jurisdictions in indication guidance for lung, CRC and oesophagogastric cancers

Cancer site	Similarities	Differences
**Lung**	All jurisdictions recommend the use of PET-CT in staging NSCLC in patients who are potential candidates for curative surgery or radical treatment.	Use of PET-CT in assessing recurrence for NSCLC varies, it is recommended in 6/13 jurisdictions (Wales, Scotland, Australia, England, Nova Scotia and Alberta). No specific mention of SCLC except for guidelines in Wales, England, New Zealand and Ontario; they recommend PET-CT in staging of limited-disease SCLC where radical therapy is being considered.
**CRC**	No ICBP jurisdictions recommend PET-CT in the initial staging of CRC, unless metastases evident at first presentation. 11 jurisdictions recommend using PET CT to re-stage after recurrence, or to assess stage ahead of liver/lung metastases surgical resection. Recurrence evaluation was the most frequent indication for PET-CT for CRC, with 10 jurisdictions incorporating this into their guidelines.	PET-CT is recommended only in England, Wales, Australia, Norway, Alberta, Ontario and New Zealand for assessing the cause of rising tumour markers when conventional imaging has shown to be negative or equivocal.
**Oesophagogastric**	12 jurisdictions recommend PET-CT for primary staging of oesophageal and/or GOJ cancers when patients are being considered for surgery or active therapy.	Variation in PET-CT indication guidance when specifying between oesophageal, gastric and GOJ cancers. Variation in the lack of direct guidelines recommending PET-CT use in gastric cancer. Only Ireland specifies using PET-CT to assess treatment response in gastric cancer patients

## Discussion

Improving the quality of cancer care, and healthcare systems more broadly, is a priority for many countries worldwide [12]. However, many ICBP jurisdictions still report a limited ability to track and evaluate the quality of care given to people with cancer. This study is an important first step in understanding whether variation in PET-CT service provision may have bearing upon cancer outcome variation internationally. Importantly, this study highlights areas of further exploration for researchers and policymakers to help optimize PET-CT services and improve the quality of cancer services.

### Variation in data accessibility and availability

Capture and analysis of good data is crucial in enabling improvements in health systems and will allow countries to learn from others’ best practice [13]. The findings from this study indicate that diagnostic data, specifically for PET-CT, is not uniformly captured, coded or accessibly stored across ICBP countries. Several factors played a role in the difficulties accessing and using PET-CT data including, but not limited to, inconsistent data definitions, data linkage issues, uncertain coverage of data and lack of specific coding e.g. purpose of the scan. Although this impacts upon how robustly we can make this type of international comparison, it enables us to use the findings from this study as a platform to call for greater, richer, more consistent data capture relating to PET-CT services globally.

Most imaging diagnostics are used for a much wider range of indications compared to PET-CT, and whilst efforts are underway to improve diagnostics data quality, data that link purpose of the diagnostic test to its activity is not readily available in most ICBP countries. This linkage is crucial in order to accurately perform benchmarking research and would be further supported by one single validated international standard for the collection and coding of PET-CT data [14]. We highlight that further research and efforts are required to progress towards this international alignment and consolidation of data capture and coding within diagnostics.

This is crucial for future research aiming to understand the relationship between the quality and capacity of PET-CT services and cancer outcomes. We have been unable to draw conclusions upon this relationship, but by improving and standardizing data capture and accessibility internationally, future research may be able to perform more in-depth analyses into this area.

### Variation in scanner quantity and acquisition over time

Previous literature validates the growth in PET-CT scanner quantity and use globally reported here [5]. Our findings further add to the evidence by demonstrating that growth in scanner and scan quantity is variable and inconsistent internationally. Denmark had the greatest quantity of scanners (0.66 per 100 000), and the greatest acquisition over time (0.02 per 100 000 in 2007 to 0.66 per 100 000 in 2017; Figure 1). Wales comparably had the lowest quantity and increase in acquisition over the same period (0 scanners in 2007; 0.04 per 100 000 in 2017). The growth in Denmark is reflective of changes brought about by the 2007 National Danish Invitation to Tender for Delivery of Cancer Scanners, as well as the introduction of national integrated pathways for cancer care [15]. Similarly, from 2004 the Norwegian Directorate of Health ordered six health technology assessments in order to increase PET-CT provision [16]. This combined with the efforts put into establishing 28 cancer patient pathways during 2015 are likely to have supported the increases in the quantity and use of PET-CT scanners and examinations (0.04 per 100 000 in 2006–09 period to 0.19 per 100 000 in 2014–17 period) [17]. Recent ICBP research demonstrated improvements in cancer survival in Denmark, for example from 27.5% 1-year lung cancer survival (1995–1999) to 46.2% (2010–2014) [1]. This will have been influenced by a multitude of factors—one of which may well be the increase in capacity of PET-CT services, and subsequently more accurate staging and treatment planning [18, 19]. However, as this relationship is not observed uniformly across all ICBP jurisdictions, it is not possible to identify clear associations and draw robust conclusions between improvements in PET-CT services and improved cancer outcomes. Lack of resources in cancer care has previously been reported to impact upon cancer outcomes, however it is not yet clear whether variability in capacity of services may do so as well [19].

### Variation in clinical indication guidance

This study has also demonstrated that variation exists in PET-CT clinical indication guidance between ICBP countries, a finding not previously reported to our knowledge. The differences seen in international indication guidance may mean the processes and resources are not in place for clinicians to refer cancer patients for PET-CT scans they could benefit from. This was particularly evident within recommendations for staging of small-cell lung cancer (SCLC) and within the specification for use in gastric cancer. Despite the variability around these areas, the evidence for the benefit of utilizing PET-CT in these patients is growing. This study provides insights to develop further in-depth studies investigating how international indication guidance responds to the current literature upon the best use of PET-CT.

Indications for the use of PET-CT in NSCLC is likely to reflect the strong evidence supporting use in staging these patients [20]. Similar consistency is not seen for SCLC internationally, with only Wales, England, Ontario and New Zealand identifying PET-CT to be used in staging limited-disease SCLC when considering curative treatment. Accurate staging in SCLC is key to ensure combined modality treatment (chemotherapy with radiotherapy) is only offered to limited-disease patients where it is most effective [21].

PET-CT is an expensive modality, and it is essential that indication guidance is not only built upon the strongest clinical evidence, but with the confidence that it is cost-effective, through the reduction of unnecessary treatments as mentioned [22]. Cost analyses have been undertaken investigating incorporating PET-CT into SCLC management in Australia and the USA, both of which demonstrated no increased cost for limited-disease management, with potential cost reduction through avoiding futile radiotherapy [21, 23].

Gastric cancer was generally grouped with oesophagogastric cancers across ICBP countries, with only Ireland specifically mentioning gastric cancer within their guidance. Oesophageal and gastro-oesophageal junction (GOJ) cancers sit within oesophagogastric cancers, yet have similar tumour biology and treatment, with gastric cancer sitting separately [24]. There has been a lack of consensus for using PET-CT in gastric cancer, with evidence demonstrating its benefit in patient management when the patient population is carefully selected. It has been shown to have greater specificity and sensitivity, and beneficially influence patient management in intestinal subtype cancer, in early stage and where tumours have a high baseline FDG uptake [25]. Evidence shows PET-CT identifying originally overlooked metastases in gastric cancer patients, leading to clinically important changes in treatment [26]. With this lack of specificity in indication guidance, it is not possible to identify how many gastric cancer patients may be missing out upon a possibly beneficial PET-CT scan. This should be further investigated in line with the clinical utility of PET-CT within this indication.

The variability seen in ICBP PET-CT clinical guidance demonstrates that there needs to be continued development of the indication evidence base for PET-CT, but also the development of the means to measure and assess how the current evidence base is incorporated into guidelines internationally. Optimization of cancer services does not simply require increasing the number of PET-CT scanner and scans, rather careful consideration as to the optimal incorporation of PET-CT into cancer pathways reflecting on impacts to timeliness of care, cost-effectiveness and cancer outcomes [27]. With the lack of easily accessible and coordinated data on PET-CT use internationally, it is not possible to draw robust conclusions regarding the specific use of PET-CT within approved indications. This further supports the need for uniform improvements in data practices globally.

## Limitations

Limitations of the study include missing PET-CT metric data and variability of data resources. Definitions on data metrics were provided to stakeholders, but we note there may be variability within individual interpretation of these. It is possible that duplicate counting may have occurred in data collected upon number of scans occurring in the general and cancer-site-specific populations. This may be the case particularly in Canada, whereby the referrals from PEI may have been counted twice depending upon the centre PEI referred each patient to, though the impact of this is likely to be small due to the small population size and subsequent incidence of cancer (in 2015, approximately 910 residents of PEI were diagnosed with cancer, with a 380 estimated to die from cancer [28]).

There is inherent variability in jurisdictional approaches to diagnostic work up regarding investment profiles for diagnostic services, and preferences for which imaging modality to refer patients to. Whilst we focus on PET-CT as just one proxy for investment in cancer services, we acknowledge that funding priorities differ between jurisdictions and precedent may be taken by another diagnostic modality for investment.

We were unable to perform more robust modelling of the factors relevant to PET-CT and cancer patient outcomes, which restricted our ability to comment in depth about the direct impact of international variation in PET-CT service provision upon the quality of cancer care and subsequent patient outcomes.

## Conclusion

This study provides a significant and international overview of PET-CT services and activity up to date. Variation in PET-CT scanner quantity, acquisition over time and guidance upon use exists internationally, but there is limited and inconsistent data capture and accessibility of PET-CT service data. We demonstrate the variation in clinical indication guidance and that further research is required to understand the impact of this variation upon cancer outcomes. This study lays the foundation for more robust in-depth analyses where the data exist and could go further by linking data sets and expanding information sources used. More work is needed to develop an assessment of the means to monitor the incorporation of clinical indication evidence regularly into international guidance. To better understand the relationship between service provision and cancer outcomes, there needs to be a movement for greater, richer data capture and coding of PET-CT services globally. This will help facilitate effective monitoring of services in order to optimize PET-CT scanning and cancer services globally.

## Supplementary Material

mzaa166_Supp

## Data Availability

The data underlying this article will be shared on reasonable request to the corresponding author.

## References

[1] Arnold M , Rutherford MJ, Bardot A et al. Progress in cancer survival, mortality, and incidence in seven high-income countries 1995–2014 (ICBP SURVMARK-2): a population-based study. *Lancet Oncol* 2019;20: 1493–505. 31521509 10.1016/S1470-2045(19)30456-5 PMC6838671

[2] Czernin J , Allen-Auerbach M, Nathanson D et al. PET/CT in oncology: current status and perspectives. *Curr Radiol Rep* 2013;1:177–90. 24883234 10.1007/s40134-013-0016-x PMC4034170

[3] Xu G , Zhao L, He Z. Performance of whole-body PET/CT for the detection of distant malignancies in various cancers: a systematic review and meta-analysis. *J Nucl Med* 2012;53:1847–54. 23073605 10.2967/jnumed.112.105049

[4] Farwell MD , Pryma D, Mankoff DA. PET/CT imaging in cancer: current applications and future directions. *Cancer* 2014;120:3433–45. 24947987 10.1002/cncr.28860

[5] Buck A , Hermann K. Economic evaluation of PET and PET/CT in oncology: evidence and methodologic approaches. *J Nucl Med Technol* 2010;38:6–17. 20197541 10.2967/jnmt.108.059584

[6] Dhingra V , Mahajan A, Basu S. Emerging clinical applications of PET based molecular imaging in oncology: the promising future potential for evolving personalized cancer care. *Indian J Radiol Imaging* 2015;25:332–41. 26752813 10.4103/0971-3026.169467 PMC4693380

[7] Neal RD , Tharmanathan P, France B et al. Is increased time to diagnosis and treatment in symptomatic cancer associated with poorer outcomes? Systematic review. *Br J Cancer* 2015;112:92–107. 10.1038/bjc.2015.48 PMC4385982 25734382

[8] Tørring M , Falborg A, Jensen H et al. Advanced-stage cancer and time to diagnosis: an International Cancer Benchmarking Partnership (ICBP) cross-sectional study. *Eur J Cancer Care (Engl)* 2019;28:e13100. 10.1111/ecc.13100 31119836

[9] Hamilton W , Green T, Martins T et al. Evaluation of risk assessment tools for suspected cancer in general practice: a cohort study. *Br J Gen Pract* 2013;63:30–6. 10.3399/bjgp13X660751 PMC3529290 23336455

[10] International Atomic Energy Agency . *Strategies for Clinical Implementation and Quality Management of PET Tracers*. Vienna: IAEA, 2009.

[11] Raghupathi W , Raghupathi V. Big data analytics in healthcare: promise and potential. *Health Information and Science Systems* 2014;2: 2047–501. 10.1186/2047-2501-2-3 PMC4341817 25825667

[12] Prager GW , Braga S, Bystricky B et al. Global cancer control: responding to the growing burden, rising costs and inequalities in access. *ESMO Open* 2018;3:e000285. 10.1136/esmoopen-2017-000285 PMC5812392 29464109

[13] Kruse CS , Goswamy R, Raval Y et al. Challenges and opportunities of big data in health care: a systematic review. *JMIR Med Inf* 2016;4:e38. 10.2196/medinform.5359 PMC5138448 27872036

[14] Ellis DW , Srigley J. Does standardised structured reporting contribute to quality in diagnostic pathology? The importance of evidence-based datasets. *Virchows Arch Eur J Pathol* 2016;468:51–9. 10.1007/s00428-015-1834-4 26316184

[15] Hoilund-Carlsen PF , Gerke O, Vilstrup MH et al. PET/CT without capacity limitations: a Danish experience from a European perspective. *Eur Radiol* 2011;21:1277–85. 21274717 10.1007/s00330-010-2025-y PMC3088822

[16] Norum J , Søndergaard U, Traasdahl E et al. PET-CT in the sub-arctic region of Norway 2010–2013. At the edge of what is possible? *BMC Med Imaging* 2015;15. 10.1186/s12880-015-0073-0 PMC4551695 26316132

[17] Haland E , Melby L. Keeping up with the codes; accounting for quality in cancer patient pathways (CPPs) in Norway. *Eur J Public Health* 2019;29:522.

[18] Petersen H , Nielsen M, Hoilund-Carlsen M et al. PET/CT may change diagnosis and treatment in cancer patients. *Dan Med Bull* 2010;57:A4178. 20816018

[19] Wait S , Han D, Muthu V et al. Towards sustainable cancer care: reducing inefficiencies, improving outcomes—a policy report from the All. Can initiative. *J Cancer Policy* 2017;13:47–64.

[20] Volpi S , Ali J, Tasker A. The role of positron emission tomography in the diagnosis, staging and response assessment of non-small cell lung cancer. *Ann Transl Med* 2018;6:95. 10.21037/atm.2018.01.25 PMC5890043 29666818

[21] Ruben J , Ball D. The efficacy of PET staging for small-cell lung cancer: a systematic review and cost analysis in the Australian setting. *J Thoracic Oncol* 2012;7:1015–20. 10.1097/JTO.0b013e31824fe90a 22534816

[22] Saif MW , Tzannou I, Makrilia N et al. Role and cost effectiveness of PET/CT in management of patients with cancer. *Yale J Biol Med* 2010;83:53–65. 20589185 PMC2892773

[23] Zer A , Gordon N, Duszak R et al. FDG-PET/CT in small cell lung cancer: a value analysis. *J Clin Oncol* 2017;34:6626–6626.

[24] Barra WF , Moreira FC, Cruz AMP et al. GEJ cancers: gastric or esophageal tumors? Searching for the answer according to molecular identity. *Oncotarget* 2017;8:104286–94. 29262640 10.18632/oncotarget.22216 PMC5732806

[25] Bosch K , Chicklore S, Cook G et al. Staging FDG PET-CT changes management in patients with gastric adenocarcinoma who are eligible for radical treatment. *European Journal of Nuclear Medicine and Molecular Imaging* 2019;47:759–67. 31377821 10.1007/s00259-019-04429-x PMC7075833

[26] Findlay JM , Antonowicz S, Segaran A et al. Routinely staging gastric cancer with F-FDG PET-CT detects additional metastases and predicts early recurrence and death after surgery. *Eur Radiol* 2019;29: 2490–8. 30643947 10.1007/s00330-018-5904-2 PMC6443603

[27] Gomez DR , Liao K, Swisher SG et al. Time to treatment as a quality metric in lung cancer: staging studies, time to treatment, and patient survival. *Radiother Oncol* 2015;2:115. 10.1016/j.radonc.2015.04.010 26013292

[28] PEI H . *PEI Cancer Strategy*. Charlottetown: Health PEI, 2016.

