# Supplementary material for: A comparative analysis: international variation in PET-CT service provision in oncology—an International Cancer Benchmarking Partnership study
Source: Int J Qual Health Care. 2020 Dec 30;33(1):mzaa166. doi: 10.1093/intqhc/mzaa166 (PMC7896108; doi:10.1093/intqhc/mzaa166)
Supplement: mzaa166_Supp [file mzaa166_supp.zip › Supplementary Materials - ICBP PET-CT.docx]

**Supplementary Materials**

**Table 1. Impact of PET-CT on staging in NSCLC**

| **Ref (year, country)** | **Study aim** | **N of patients** | **Key findings** |
| --- | --- | --- | --- |
| Bury et al. (1997, Belgium) | Compare accuracy of PET and conventional imaging for staging of NSCLC | 109 | PET changed overall stage 34%; N stage changed in 20% patients and M stage in 14% |
| Saunders et al. (1999, UK) | Assess impact and prognostic significance of PET in staging of patients considered suitable for surgery | 97 | PET correctly altered stage in 27%; changed N stage in 13%; detected distant metastases in 17% |
| Pieterman et al. (2000, Netherlands) | Compare PET with conventional imaging in detecting lymph node and distant metastases in NSCLC patients | 102 | PET changed stage in 60.7% - upstaged in 41.2%, downstaged in 19.6% |
| Hicks et al. (2001, Australia) | Evaluate impact of PET in lung cancer staging on patient treatment and outcomes | 153 | PET changed overall stage by 43%; 10% downstaged, 33% upstaged; 22% patients treatment changed from curative to palliative |
| Hoekstra et al. (2003, Netherlands) | Assess value of PET in staging stage IIIA N2 NSCLC patients when considering combined modality therapy | 57 | PET suggested upstaging in 56% which was confirmed in 30% |
| Antoch et al. (2003, Germany) | Determine accuracy of PET-CT compared with PET alone and CT alone in NSCLC staging | 27 | PET-CT changed staging in 15% PET alone and 19% in CT alone |
| Gregory et al. (2012, Australia) | Investigate management impact and prognostic value of staging with PET-CT in NSCLC patients | 168 | PET-CT altered staging in 50.6% patients with 41.1% upstaged, 9.5% downstaged; management impact in 42.3% patients |
| Takeuchi et al. (2014, Spain) | Determine impact of initial PET-CT staging on clinical stage, management plan and prognostic value in NSCLC | 592 | PET-CT changed stage in 28.7% patients; 16.4% upstaged, 12.3% downstaged; impacted 37.2% management plans |
| Taus et al. (2014, Brazil) | Investigate the impact of PET-CT on the therapeutic management of NSCLC patients | 246 | In 34.6% cases, PET-CT led to stage migration; treatment modified in 24.4%; futile thoracotomies avoided in 5.2% |
| Zheng et al. (2014, China) | Analyse use of PET-CT for staging and evaluate impact on radiotherapy volume delineation compared with CT in NSCLC patients | 23 | PET-CT changed TNM staging in 35% cases; alterations in radiotherapy planning in 60% |
| Geiger et al (2014, USA) | Examine rates of tumour progression in NSCLC patients as determined by repeat treatment planning PET-CT | 47 | New nodal or metastatic disease identified by PET-CT in 51% patients; upstaging rate 17% |

**Table 2. PET-CT Access Metric Data Sources**

| **Jurisdiction** | **Data source** |
| --- | --- |
| **Australia**  *New South Wales*  *Victoria*  *Western Australia* | **N/A** No appropriate data source identified – location of scanners identified through Medicare Benefits Schedule Australia |
| **Canada**  *Alberta*  *New Brunswick^1^*  *Nova Scotia^1^*  *Ontario ^1^*  *Prince Edward Island*  *Saskatchewan* | Cerner Millennium (Calgary); Agfa RIS (Edmonton)  PEI Physician's Billing database  Montage database |
| **Denmark** | Danish Lung Cancer Group Annual Reports |
| **Ireland** | National Clinical Programme for Radiology (NCPR); Irish Nuclear Medicine Association (INMA) |
| **New Zealand** | COMRAD software (Christchurch PET-CT centre only) |
| **Norway** | RIS/PACS system |
| **UK**  *England*  *Northern Ireland*  *Scotland*  *Wales* | IC10 code database; Radiology information system (RIS)  Royal Victoria Radiology Information system  National Radiology Information and Intelligence Platform  RADIS database – local Radiology information system |

*^1^Data source not provided by jurisdictional contacts*

**Table 3. Data availability on PET-CT access metrics in ICBP jurisdiction**

| **Jurisdiction** | **N of scans (general pop)** | **N of scans (cancer pop)** | **N of scans per cancer site** | **Wait time** | **Cost of scanner** | **Cost of scan** | **Cost of FDG dose** |
| --- | --- | --- | --- | --- | --- | --- | --- |
| Alberta | Y | Y | Y | Y | Y | Y | Y |
| Australia | N | N | N | N | N | N | N |
| Denmark | N | N | Y^1^ | N | N | N | N |
| England | Y^2^ | Y^2^ | Y^2^ | Y^2^ | N | N | N |
| Ireland | Y^3^ | N | N | Y^3^ | Y | Y | N |
| New Brunswick | Y | Y | N | N | Y | Y | N |
| New Zealand^4^ | Y | Y | Y^5^ | Y | N | Y | Y |
| Northern Ireland | Y | Y | Y | N | Y | Y | N |
| Norway | Y^6^ | Y^7^ | N | Y^6^ | N | N | N |
| Nova Scotia | Y | Y | Y | N | Y | N | N |
| Ontario | Y | Y | Y | Y | N | Y | Y |
| Prince Edward Island | Y | Y | Y | N | N | Y | N |
| Saskatchewan | Y | N | Y | N | Y | Y | N |
| Scotland^7^ | Y | Y | Y | Y | Y | Y | Y |
| Wales | Y | Y | Y | Y | Y | Y | Y |

*^1^Lung cancer only*

*^2^Incomplete data – only from alliance medical scanners and 2 additional scanner centres*

*^3^2016-2018 only*

*^4^Data only available from 1 out of 5 New Zealand scanner centres*

*^5^Limited data with varying years available*

*^6^Varying levels of data from each centre with varying dates available*

*^7^Limited number of scanner centres with varying dates covered*

**Table 4. Confirmed Clinical Indications for ICBP cancer sites of interest**

*N.B indication guidance is relevant within study dates; please note these may have been developed further since 2017*

| **Jurisdiction** | **Source** | **Lung** | **CRC** | **Oesophagogastric** | **Ovarian** | **Pancreatic** | **Liver** |
| --- | --- | --- | --- | --- | --- | --- | --- |
| England | RCR 2016 Guidelines | Differentiation of benign vs malignant lesions where anatomical imaging/biopsy inconclusive.  Staging for NSCLC and limited SCLC pts considered for radical treatment (curative intent).  Characterisation of SPN with risk of malignancy <10%.  Pre-op staging of NSCLC. Assessing recurrent disease where anatomical imaging inconclusive. Assessing response to chemo/radio therapy when considering surgery. Differentiate recurrence and treatment effects. | Stage pts with suspected liver/lung metastases on other imaging.  Restage pts with recurrence considered for radical treatment.  Scan prior to metastectomy.  Assess recurrent disease (rising markers)  Assess response in rectal carcinoma post chemo/RT with indeterminate findings on other imaging.  Assess response post liver/lung metastases ablation. | Staging primary cancer^1,2^.  Assess disease recurrence in previously treated oesophageal cancer.  Evaluation of suspected recurrence when other imaging equivocal^2^ | Detection of tumour in selected patients with rising CA 125 levels and equivocal imaging.  In difficult management situations to assess local and distant spread. | ^5^ | ^5^ |
| Wales | Welsh Health Specialised Services Committee | Investigating NSCLC patients who are candidates for radical RT/surgery  Investigation of SPN when biopsy has failed or not possible.  Assessing recurrence for NSCLC when radical treatment considered; Assessing extent of mesothelioma prior to radical decortication. | Restaging of CRC prior to surgery to remove liver/lung metastases.  Restaging when conventional imaging failed to show cause of rising tumour markers. | Staging prior to radical treatment^1^. | ^5^ | ^5^ | ^5^ |
| Northern Ireland | Northern Ireland Cancer Network Guidelines | Staging of NSCLC when considering surgery/radical RT.  Staging of SCLC in specific clinical circumstances.  Assessment of SPN. Assessment of response to first-line therapy in NSCLC. | Stage patients at high risk of liver metastasis (e.g. T4 perforated). Pre-op/post-op scanning when metastases suspected. Can be considered when elevated tumour marker level with negative/equivocal CT; distinguishing recurrence. | **^5^** | Not recommended for ovarian carcinoma. | Can be used in staging where conventional imaging has been equivocal for metastases and presence of metastases would lead to a decision not to operate. | Can be used to indicate success of ablation at an early stage.  Exclude presence of metastases in cholangiocarcinoma in additional to conventional treatment plan. |
| **Jurisdiction** | **Source** | **Lung** | **CRC** | **Oesophagogastric** | **Ovarian** | **Pancreatic** | **Liver** |
| Scotland | Scottish Clinical Imaging Network Guidelines 2016 | Staging of stage I-III NSCLC when radical RT proposed.  Evaluating recurrence when conventional imaging inconclusive.  Assessing SPN when biopsy risky or non-diagnostic.  Assessing solitary site of possible metastatic disease. | Patients with liver/lung metastases being considered for resection.  All patients with mCRC being considered for surgery or radical therapy. | For pts deemed suitable for radical concurrent chemoradiotherapy or surgery^1^.  Detection of recurrent disease when conventional imaging equivocal^2^. | ^5^ | ^5^ | ^5^ |
| Ireland | RCSI; Mater Miseriocordiae University HOspital referral guidelines | Initial staging of lung cancer.  Diagnosis of cancer in SPN.  Diagnosis of primary lesion when multiple pulmonary nodules present.  Staging prior to resection of solitary lung metastasis.  Response to treatment evaluation – post-radical RT or ablation. | Restaging of known liver metastasis pre-resection to exclude extrahepatic disease.  Check if recurrent disease is localised.  Restaging with clinical suspicion of recurrence with negative/equivocal CT with normal or elevated antigen level.  Response evaluation to treatment if change in treatment contemplated. | Response evaluation post-chemo when conventional imaging equivocal^1,4^; Assess recurrence where CT equivocal^2;^; Initial staging^2^; Restaging after neoadjuvant therapy when considered for curative resection^2^ | Staging with rising marker or suspected recurrence with negative or equivocal CT. | Use in selected cases after endoscopic ultrasound and CT evaluation, following MDT discussion. | ^5^ |
| **Jurisdiction** | **Source** | **Lung** | **CRC** | **Oesophagogastric** | **Ovarian** | **Pancreatic** | **Liver** |
| Australia | Cancer Council Australia | Staging of proven NSCLC when curative RT or surgery is planned.  Staging following pathological confirmation of NSCLC.  Guide RT planning.  Pre-treatment metastatic assessment (NSCLC).  Evaluation of malignancy in indeterminate SPN.  Assessing suspected recurrence.  To determine treatment effectiveness. | Detect additional metastases in patients with potentially resectable lung and liver metastases.  In suspected residual, metastatic or recurrent CRC in patients where active therapy is being recommended. | Staging when considered for active therapy^1^.  Staging of regional & distant metastases^1^.  Pre and post-treatment assessment of metastatic disease^1^.  Assess effect of neoadjuvant chemoradiotherapy^1^.  Assess suspected recurrence^1^. | Initial staging for distant metastases to guide treatment.  Evaluate suspected residual, metastatic, recurrent carcinoma following initial therapy. | ^5^ | ^5^ |
| New Zealand | Southern Cross Health Society and Southern Network District Health Board (DHB) | Initial staging (NRV); Staging for pts with limited SCLC when considered for radical therapy.  Staging of NSCLC prior to surgery/radiotherapy (curative intent); For isolated pulmonary nodules not amenable to FN (NSCLC). | Staging of histologically proven recurrent CRC where pelvic exenteration being considered; Pre-op evaluation for resection of liver/lung metastases.; Restaging for metastatic disease. | Staging of locally  advanced cancer^1^.  Staging of oesophageal/gastric for curative treatment. | Restaging of recurrence when curative surgery/therapy considered. | ^5^ | ^5^ |
| **Jurisdiction** | **Source** | **Lung** | **CRC** | **Oesophagogastric** | **Ovarian** | **Pancreatic** | **Liver** |
| Nova Scotia | TRIUMF report on use of PET across Canada | Diagnosis of SPN.  Restaging for recurrences.  Distant staging.  Disease/therapeutic monitoring. | Evaluation of recurrence.  Restaging & investigating distant metastases.  Disease/therapeutic monitoring. | Staging^3^  Monitoring of oesophageal cancer.  Restaging of oesophageal. | ^5^ | ^5^ | ^5^ |
| Alberta | TRIUMF report on use of PET across Canada | Diagnosis.  Staging.  Investigation of SPN.  Pre-surgical assessment and for radiotherapy planning.  Restaging/local recurrences.  Therapy/disease monitoring.  Mesothelioma – staging, restaging, recurrence, presurgical assessment, RT planning. | Staging after recurrence.  Pre-surgical assessment.  Radiotherapy planning.  Investigate distant metastases and persistent elevated tumour marker levels. | Primary staging^1^.  Presurgical assessment of oesophageal cancer  Radiotherapy planning^3^  For distant metastases^4^.  Restaging/local recurrence^3^.  Therapy/disease monitoring^1^. | Assess surgical resectability.  Investigate elevated tumour markers.  Staging after recurrence. | ^5^ | ^5^ |
| Ontario | Cancer Care Ontario PET guidelines | Staging I-III NSCLC when potential curative treatment being considered. Restaging NSCLC when locoregional recurrence after primary treatment.  Staging limited SCLC when combined modality therapy being considered.  Investigation of SPN when biopsy unsuccessful.  Staging mesothelioma | Staging/restaging of limited metastatic CRC and of limited local recurrence when considered for radical intent therapy.  When recurrence suspected due to rising tumour markers but imaging tests negative or equivocal. | Baseline staging for patients being considered for curative therapy^2^.  Repeat PET-CT on completion of pre-surgery neoadjuvant therapy^3.^  Restaging of locoregional recurrence after primary treatment^1^. | Re-staging of recurrent gynaecological malignancies under consideration for radical salvage surgery. | ^5^ | ^5^ |
| Saskatchewan | Saskatchewan PET Programme – Approved Indications for FDG-PET 2013 | Staging of NSCLC with stage I, IIa lesions.; Staging of potentially resectable stage IIb and III NSCLC; Planning radical RT for NSCLC; Staging prior to resection of solitary lung metastasis; Characterisation of SPN. | Staging in patients with potentially resectable recurrence in colon/rectal cancer. | Baseline evaluation for oesophagectomy; Restaging after neoadjuvant chemotherapy and/or RT for pts considered for radical resection; | ^5^ | ^5^ | ^5^ |
| Denmark | Danish Health Authority Cancer Guidelines, DMCG Clinical Guidelines | Staging of NSCLC and in patients who can potentially receive curative treatment.  Pre-op scan to detect metastases. | Staging advanced & recurrent rectal cancer; Pre-operative staging; Post-treatment. | Primary staging when candidates for surgery^1^ | Routine staging & prior to surgery. | Can be used as a supplement in pre-therapeutic  Staging. | Pre-op staging for suspected extrahepatic dissemination. |
| **Jurisdiction** | **Source** | **Lung** | **CRC** | **Oesophagogastric** | **Ovarian** | **Pancreatic** | **Liver** |
| Norway | Norwegian Health Authority, Cancer Guidelines | Staging of NSCLC and in patients who can potentially receive curative treatment. | ^5^ | Baseline and restaging after neoadjuvant chemotherapy and/or RT for pts considered for radical resection.  Delineation of gross tumour volume in pts receiving RT. | Can be used as a supplement in pre-therapeutic  staging or recurrence | Can be used as a supplement in staging where conventional imaging has been equivocal or for metastases and presence of metastases in patients with potentially respectable disease. | Pre-op staging for patients with suspected extrahepatic dissemination and before liverTX |

*^1^For oesophageal/gastro-oesophageal junction cancers.*

*^2^For oesophageal and oesophagogastric cancers.*

*^3^For oesophageal cancer only.*

*^4^For gastric cancer only*

*^5^No publicly available appropriate indication source identified*

**Table 5. Number of scans per 100,000 in cancer population 2010-17**

| Year | Alberta | New Brunswick | Northern Ireland | Nova Scotia | Ontario | Wales | *PEI^1^* |
| --- | --- | --- | --- | --- | --- | --- | --- |
| 2010 | - | 126 | 106 | 170 | 49 | - | *32* |
| 2011 | 45 | 141 | 112 | 177 | 58 | 56 | *53* |
| 2012 | 62 | 163 | 123 | 196 | 64 | 62 | *49* |
| 2013 | 67 | 167 | 142 | 225 | 70 | 71 | *61* |
| 2014 | 81 | 184 | 154 | 239 | 79 | 84 | *67* |
| 2015 | 82 | 192 | 159 | 236 | 86 | 93 | *62* |
| 2016 | 119 | 220 | 164 | 252 | 93 | 100 | *61* |
| 2017 | 119 | 242 | 154 | 260 | 103 | 102 | *89* |
| % increase^2^ | 164.4% | 92.1% | 45.3% | 52.9% | 110.2% | 82.1% | *178.1%* |
| Factor increase^3^ | 2.6 | 1.9 | 1.5 | 1.5 | 2.1 | 1.8 | *2.8* |

*N.B No or incomplete data available for Australia, Denmark, Norway, England, New Zealand, Scotland*

*^1^PEI data is number of referrals out of province for PET-CT scan.*

*^2^Percentage increase calculated from 2010 to 2017 for NI, Ontario, New Brunswick, PEI, Nova Scotia; increase calculated from 2011 to 2017 for Wales and Alberta*

*^3^For every 1 scan in 2010, there are now X in 2017*
